# Spatiotemporal occupancy patterns of chronic wasting disease

**DOI:** 10.3389/fvets.2024.1492743

**Published:** 2024-11-20

**Authors:** Amy J. Davis, Shane Hesting, Levi Jaster, Joseph E. Mosley, Akila Raghavan, Ram K. Raghavan

**Affiliations:** ^1^National Wildlife Research Center, Animal and Plant Health Inspection Service, United States Department of Agriculture, Fort Collins, CO, United States; ^2^Kansas Department of Wildlife and Parks, Emporia, KS, United States; ^3^Department of Veterinary Pathobiology, College of Veterinary Medicine, University of Missouri, Columbia, MO, United States; ^4^Department of Public Health, College of Health Sciences, University of Missouri, Columbia, MO, United States

**Keywords:** chronic wasting disease, occupancy modeling, environmental risk, Kansas, spatiotemporal, Bayesian, white-tailed deer, mule deer

## Abstract

**Introduction:**

Chronic wasting disease (CWD) among cervids in Kansas has seen a consistent rise over the years, both in terms of the number of infections and its geographical spread. In this study, we assessed the occupancy patterns of CWD among white-tailed deer and mule deer across the state.

**Methods:**

Using surveillance data collected since 2005, we applied a dynamic patch occupancy model within a Bayesian framework, incorporating various environmental covariates. Using principal components analysis, 13 fully orthogonal components representing cervid habitat, soil, and elevation were derived. Competing models with different temporal patterns were fit, and the best model selected based on Watanabe-AIC values and AUC value of 0.89.

**Results:**

The occupancy pattern produced by this model revealed a steady progression of the disease toward the east and southeast of the state. A random forest analysis of covariates at annual intervals indicated that geographic location, elevation, areas occupied by mixed forests, and several soil attributes (pH, clay content, depth to restrictive layer, available water content, and bulk density) explained most of the variability in the surveillance data (*R*^2^ = 0.96).

**Discussion:**

The findings reported in this study are the first for the state of Kansas but are consistent with previous findings from other geographic jurisdictions in the US and Canada. This consistency underscores their value in designing surveillance and management programs.

## 1 Introduction

Chronic wasting disease (CWD) is a neurodegenerative disease of cervids caused by a misfolded prion protein (PrP^CWD^), through contact with other infected deer, from the environment as well as transmitted vertically during birth (1, 2). Infected deer, including white-tailed deer (*Odocoileus virginianus*), mule deer (*O. hemionus*), elk (*Cervus canadensis*), and moose (*Alces alces*) exhibit symptoms of wasting, display behavioral changes such as decreased social interaction, loss of awareness, and loss of fear for humans, and exhibit polydipsia (increased drinking), polyuria (increased urination), and hypersalivation (excessive salivation) (3). Infection will lead to premature death, which leads to concerns for conservation as well as loss of revenue to rural economies that rely on big game hunting (4, 5). Although currently there is no evidence, there is additionally a looming concern that CWD spillover to humans could occur in the future (6, 7) as venison consumption is common among hunters and their families, which may result in human suffering similar to that seen with mad cow disease outbreak in the 1990s. Such negative perception will lead to a decline in big game hunting, which will have a serious economic impact and consequences for deer conservation and management. There are no vaccines or effective preventive options available for managing this disease among free-ranging or captive cervids at the present time.

The geographic distribution of CWD-positive cervids has steadily increased over time. First noted in Colorado in 1967 at a research captive deer facility (1), CWD-positive captive and free-ranging cervids have since been detected in at least 35 states in the US and 5 provinces in Canada, mainly in the Great Plains region (8). Independent of the North American spatial distribution, the disease has also been reported among cervids in Norway, Sweden, and Finland, with an unclear origin, and in captive cervids in South Korea, which was imported from Canada (9, 10).

The past two decades have revealed a number of factors that influence the persistence of CWD infection among cervids, particularly white-tailed deer, and these factors could potentially promote spatial spread (11–13); although, the ecology and epizootiology of this disease remains yet to be fully understood. In Kansas for instance, the focus area for the present study, CWD was first noted in the year 2005 in Cheyenne county, the northwestern most county in the state, and every successive year since then the disease has been detected among white-tailed deer and mule deer from additional counties during annual surveillance. The current potential spatial distribution extent of the disease in the state, and any of its potential drivers are not known. Predicting the spatial distribution and expansion, and determining what factors, if any, in the environment could be contributing to the geographic spread of diseases, is important for planning appropriate management and surveillance strategies and for informing policy makers and public.

As it is typical for many wildlife diseases, much of the information we currently have on CWD in Kansas is based on opportunistic surveillance efforts, which rely on samples submitted for CWD testing by volunteers, viz., hunters, taxidermists, meat processors, and wildlife biologists. Conducting spatial pattern analyses and subsequent interpretations based on such data is problematic because they are likely to be a biased representation of underlying disease spatiotemporal prevalence. Novel methods have been developed over the years that addresses some of these concerns. For a disease like CWD with a highly heterogeneous prevalence across different regions in Kansas, for which surveillance data is sparsely available and collected non-uniformly over time and space, it is ideal to use occupancy methods that are flexible enough to accommodate for spatiotemporal heterogeneities in sampling and do not rely on host ecological data such as density, distribution, or population dynamics (14, 15).

Free-ranging and farmed ungulates in N. America are highly susceptible to CWD, and since its first detection the disease has numerically increased over the years and has spread relatively rapidly to newer geographic areas (8). Despite control efforts, the disease has proven difficult to contain; possibly due to the many different ways by which it is transmitted, environmental reservoirs, including soil and potentially plant materials in which the prions can remain viable for several years (16, 17). Additionally, the regulatory factors that govern big game hunting and stakeholder, landowners’ preferences influence executable management options (18, 19). The steady, almost exponential growth in white-tailed deer populations over the past decades (20), and potentially varying levels of their genetic susceptibility (21, 22) and cervid social behavior (23, 24) could also be contributing factors. Fundamental to proper management of wildlife diseases is to understand how far the disease has spread geographically, what/if there are any discernable distributional patterns, and the potential factors that contribute to such patterns. In this effort, we evaluated these aspects for CWD in Kansas for the first-time using surveillance data collected since 2005.

In this study, we used a dynamic patch-occupancy model in a Bayesian framework to quantify the spatial distribution of CWD in Kansas. Additionally, we utilized elevation, and environmental covariates derived from the land cover/land use and soil survey in order to estimate which of these factors play a role in the spatial distribution.

## 2 Materials and methods

### 2.1 Study region

Surveillance data used in this study was collected from the state of Kansas, situated in Great Plains region, and has diverse landscape and climate that is suitable for supporting cervid populations. Although cervids were virtually extirpated in Kansas in the beginning of 1900s, through hunting regulations and natural migration from neighboring states, the state’s current deer population has been restored and is considered to be healthy. White-tailed deer, the most commonly found deer species in N. America is found throughout the state with some differences in their density, in diverse land use areas such as agricultural land as well as periurban areas. Mule deer, the second most common cervid species in Kansas, have been declining overall but remain more prevalent in the western parts of the state. Climate in Kansas is continental, with great extremes between summer and winter temperatures but only short periods of extreme hot or cold. The growing season is from mid-March to mid-September and the precipitation gradually varies from east to west, with east receiving progressively more rainfall around 40 inches annually, compared to 20 inches in the west.

Kansas lies in the center of the United States, and mainly experiences three different types of climates. A small western part of the state has a semi-arid steppe (Köppen climate classification BSk) with hot summers and cold winters. The significant eastern portion has hot and humid summers and falls under the humid continental (Köppen Dfa) type. Southeastern Kansas displays a humid subtropical type (Köppen Cfa) with mild winters.

### 2.2 Disease data

#### 2.2.1 Data collection

Surveillance for CWD positive cervids in Kansas started in 2005. Since this time, the Kansas Department of Wildlife and Parks (KDWP) has continuously surveyed cervids in the state for CWD presence; however, the area under surveillance changed in 2012–2013 period when the surveys were conducted in one of 5 contiguous regions in the state, such that a higher amount of time were spent by KDWP personnel in soliciting and collecting samples from volunteers in one of these five regions, who were compensated for submitting samples. State-wide surveillance resumed in 2020. During the “deer season” from mid-November to mid-January, tissue samples *viz.*, lymph nodes and/or obex from different cervid species, but predominantly white-tailed deer were obtained through one of many sources, *viz.*, taxidermists and meat processors. Also, samples were collected from wildlife biologists, and seasonal technicians who collected samples from roadkill deer, and from deer harvested by private hunters, who were compensated for their samples by providing free CWD testing. Diagnostic testing for CWD was performed with immunohistochemistry test.

#### 2.2.2 Data curation

**Figure 1:**
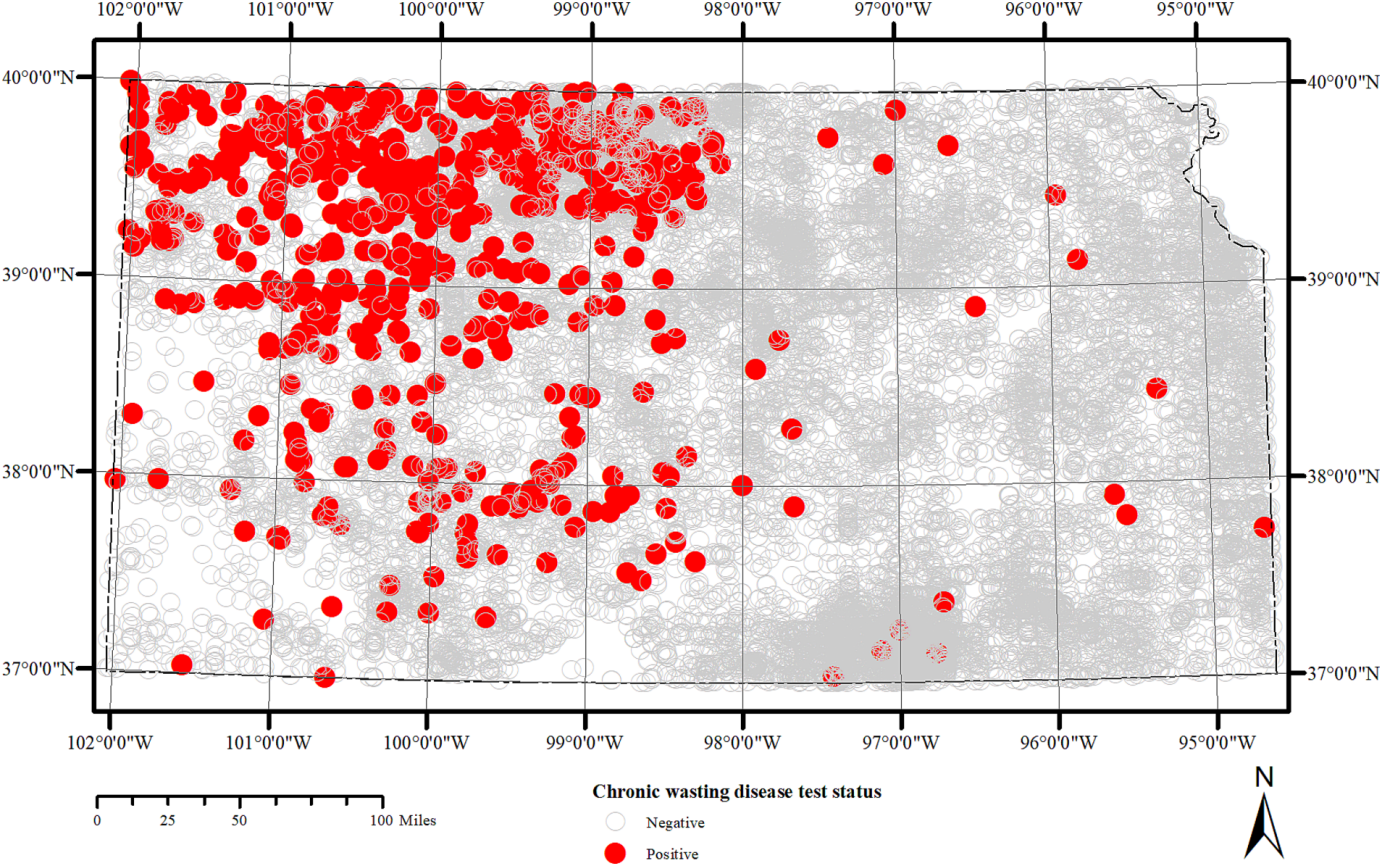
Spatial distribution of lymph node and obex samples tested for Chronic wasting disease in Kansas between 2005 and 2023. Each circle represents a sample location within 5 km radius of deer harvest.

Surveillance data from years 2005–2019 were maintained by the KDWP and data from 2019 to 2022 were collected by University of Missouri. Records in both cases were digitized from originally handwritten data on data cards that were sent by sample collectors along with tissue samples for CWD testing. The location information provided on the data card, either as geographic coordinates or verbal description were geocoded using Google Earth software. The distribution of positive and negatively tested samples submitted for CWD testing is depicted in Figure 1. Additional variables included CWD test result (positive, negative, unsuitable); age (0.5, unknown); species (white-tailed deer, mule deer); and sex (male, female, unknown). The domain values for each of these variables were standardized prior to their use in the study.

#### 2.2.3 Covariate data

Environmental characteristics such as the host’s habitat and soil properties are associated with wildlife diseases and are useful proxies for describing how the disease prevalence vary spatially. Human land use (25), land cover composition and density (26), and cervid habitat in general (27) affect CWD. The CWD prions can survive in the soil for several years and affect CWD spread (28, 29). Habitat level covariate data were obtained from two sources. The National Land cover/land use (NLCD) data was obtained from the US Geological Survey Multi-Resolution Land Characteristics MRLC consortium (30), and soil attribute data that were deemed to be relevant to CWD disease ecology were obtained from the California Soil Resource Lab, which is available in a GeoTIFF format, and are created by aggregating the USDA NRCS Soil Survey Data at the resolution of 800 m grid cells (31). Land cover/land use data were extracted within a 20 km^2^ grid. Representative values of land cover/land were the percent coverage within each grid and soil attributes were mean values within each grid. A list of land cover/land use and soil attribute data considered in this study is presented in Table 1.

**Table 1:** Environmental variables evaluated in the study.

Covariate (Data source)	Variables
Land cover/Land use (USGS, MRLC)	Open Water, Developed Open Space, Developed (Low Intensity), Developed (Medium Intensity), Developed (High Intensity), Barren, Deciduous Forest, Evergreen Forest, Mixed Forest, Shrubland, Grassland Pasture, Woody Wetlands, Herbaceous Wetlands.
Soil physiochemical properties (California Soils Resource Lab)	Calcium Carbonate (kilograms/meter^2^), Cation Exchange Capacity (centimoles/kilogram), Cation Exchange Cap. (0–5 cm) (centimoles/kilogram), Cation Exchange Cap. (0–25 cm) (centimoles/kilogram), Cation Exchange Cap. (0–50 cm) (centimoles/kilogram), Electrical Conductivity (decisiemens/meter), Electrical Conductivity (0–5 cm) (decisiemens/meter), Electrical Conductivity (0–25 cm) (decisiemens/meter), pH (0–5 cm), pH (0–25 cm), pH (25–50 cm), pH (30–60 cm), Sodium Adsorption Ratio ratio, Soil Organic Matter (kilograms/meter^2^), Soil Organic Matter—Max percent by weight, expressed as a decimal, Available Water Holding Capacity (cm), Available Water Holding Capacity. (0–25 cm) (cm), Available Water Holding Capacity. (0–50 cm) (cm), Bulk Density (1/3 bar grams/cm^3^), Drainage Class, Rock Fragments (0–25 cm), percent by volume expressed as a decimal, Saturated Hydraulic Conductivity (Ksat)—Mean (micrometers/s), Saturated Hydrologic Conductivity (Ksat)—Min (micrometers/s), Saturated Hydraulic Conductivity (Ksat)—Max (micrometers/s), Saturated Hydraulic Conductivity (Ksat) (0–5 cm) (micrometers/s), Soil Texture (0–5 cm), Soil Texture (0–25 cm), Soil Texture (25–50 cm), Sand percent by weight, Sand (0–5 cm) percent by weight, Sand (0–25 cm) percent by weight, Sand (25–50 cm) percent by weight, Sand (30–60 cm) percent by weight, Silt percent by weight, Silt (0–5 cm) percent by weight, Silt (0–25 cm) percent by weight, Silt (25–50 cm) percent by weight, Silt (30–60 cm) percent by weight, Clay percent by weight, Clay (0–5 cm) percent by weight, Clay (0–25 cm) percent by weight, Clay (25–50 cm) percent by weight, Clay (30–60 cm) percent by weight, Depth to Restrictive Layer cm, Hydrologic Group, Kw Factor (0–25 cm) factor value, Land Capability Class—Non-Irrigated class, Land Capability Class—Irrigated class, Soil Depth (cm), Soil Order, Soil Temperature Regime, Wind Erodibility Group, Wind Erodibility Index (tons/acre/year)
Elevation USGS	30 × 30 m elevation

### 2.3 Modeling

#### 2.3.1 Occupancy analysis

We used a dynamic occupancy analysis (32) to model the spatial and temporal patterns of CWD across the study area. Occupancy models jointly estimate the biological process, whether CWD was present or not (occupancy), and the observational process, the probability that CWD would be detected by the surveillance efforts if it were present (detection). We overlaid a 20 × 20 km grid across the state of Kansas, each grid represents a site (*i*). Grid sizes for occupancy analyses are often based on the average home range size for the species of interest (33). Here although the primary surveillance species was white-tailed deer, the subject of interest is CWD. We chose the grid size to balance the resolution of the surveillance data while maximizing detection probability. We used a dynamic model to account for annual variability in CWD occupancy. Our surveillance data were from annual hunting samples and therefore we used an annual temporal scale (*t*) for our analysis.

We are interested in the hidden ecological state, *z_it_*, indicating whether CWD was present in site *i* at time *t*. If *z_it_* is zero (indicating CWD was absent in site *i* at time *t*) there would be no positive detections, *y_it_*, of CWD (i.e., assuming no false positives). The gold standard diagnostic test for detecting CWD prions is immunohistochemistry, which has a negligible rate of false positives. If *z_it_* is one (indicating CWD is present in site *i* at time *t*), then the number of positive samples, *y_it_*, is a result of the total number of samples taken, *n_it_*, and the probability of detecting CWD given it is present, *p* (Equation 1). Detection probability, *p*, is modeled with an uninformative prior (Equation 2). The initial hidden ecological state of CWD presence/absence, *z_it_*, was modeled as a Bernoulli random variable with the probability of initial occupancy, *ψ_i1_* (Equation 3). The initial occupancy can be modeled as a logit transformation of a combination of covariates, *X_ψ_*, (Equation 4) and linear regression coefficients, *β_ψ_* (Equation 5). Since we used a dynamic model, the hidden state, *z_i1_*, and initial occupancy, *ψ_i1_*, were modeled separately. All subsequent hidden states, *z_it_*, were modeled conditioned on the previous time step, *z*_*it*-1_ (Equation 6) as Bernoulli random variables with probability occupancy, *ψ_it_*. All occupancies after the initial time step, *ψ_it_*, were derived from the previous state, *z*_*it*-1_, and transition parameters (Equation 7). If a site was occupied in time *t-1* (*z_it-1_* = 1), it may become unoccupied at time *t*, with the probability of local extinction, *ε_it-1_*, or stay occupied (did not go locally extinct, 1- *ε_it-1_*). If a site was unoccupied in time *t-1* (*z_it-1_* = 0), it may become occupied at time *t*, with the probability of local colonization, *γ_it-1_*, or stay occupied (1- *γ_it-1_*). Local extinction, *ε_it_*, and local colonization, *γ_it_*, can be modeled as a function of covariates (*X_ε_* and *X_γ_* respectively) and linear regression coefficients (*β_ε_* and *β_γ_* respectively; Equations 8–11). We used uninformative priors on *p*, *β*_ψ_, *βε*, and *βγ* as we did not have strong prior knowledge to help inform the model.


(1) $$y_{\text{it}}\sim \text{Binomial}\left(z_{\text{it}}*p,n_{\text{it}}\right)$$

(2) $$p\sim \text{Beta}\left(1,1\right)$$

(3) $$z_{i1}\sim \text{Bernoulli}\left(\psi_{i1}\right)$$

(4) $$\text{logit}\left(\psi_{i1}\right)=X_{\psi }\beta_{\psi }$$

(5) $$\beta_{\psi }\sim \text{Norm}\left(0,1\right)$$

(6) $$z_{\text{it}}|z_{\text{it}-1}\sim \text{Bernoulli}\left(\psi_{\text{it}}\right)$$

(7) $$\psi_{\text{it}}=\left(1-\varepsilon_{\text{it}-1}\right)*z_{\text{it}-1}+\gamma_{\text{it}-1}\left(1-z_{\text{it}-1}\right)$$

(8) $$\text{logit}\left(\varepsilon_{\text{it}}\right)=X_{\varepsilon }\beta_{\varepsilon }$$

(9) $$\beta_{\varepsilon }\sim \text{Norm}\left(0,1\right)$$

(10) $$\text{logit}\left(\gamma_{\text{it}}\right)=X_{\gamma }\beta_{\gamma }$$

(11) $$\beta_{\gamma }\sim \text{Norm}\left(0,1\right)$$

Local colonization rates were of particular interest in this analysis, and we modeled many covariate relationships associated with local colonization. We examined habitat effects using the National Land Cover Database (34) and we included a collection of soil characteristics (full set of possible covariates shown in Table 1). We modeled annual variability using splines on time from package ‘splines2’ (35), and we accounted for the proportion of neighboring sites that were occupied on the previous time step (termed neighbor effect). We subset the covariates for the occupancy analysis to limit those used to ones that were not highly correlated (*R* > 0.5), using the ‘caret’ package (36). The dynamic occupancy model has difficulty converging when there are too many predictor variables. Therefore, we chose the cutoff of 0.5 to ensure the number of predictors was less than 20. We then conducted a principal components analysis (PCA) [PCA; (37)] to create covariates for the dynamic occupancy analysis that were completely orthogonal to avoid potential issues with multicollinearity. We compared models with different temporal patterns, we examined a linear trend across years and splines on year with 3, 5, or 7 degrees of freedom. Model comparisons were conducted using Watanabe-Akaike information criterion—WAIC [(63, 38)]. WAIC is similar to AIC where the more parsimonious models are suggested with lower values. We examined goodness of fit of the model set using the area under the curve (AUC) statistic adjusted for the imperfect detection associated with occupancy models (39).

We calculated the posterior distributions for the dynamic occupancy model using a custom coded Markov Chain Monte Carlo (MCMC) algorithm with Metropolis-Hastings steps in Program R (40). We assessed convergence visually and using the Gelman-Rubin statistic (41).

#### 2.3.2 Post-hoc random forest analyses

All occupancy values after the initial occupancy were derived from the previous time steps and transition rates. We wanted to understand factors associated with the derived occupancy estimates. We used a random forest approach (42) to evaluate which of a the broader set of predictor variables (Table 1) were most able to explain the variation in the posterior estimates of occupancy. We implemented the analyses using the package ‘randomForestSRC’ (43) in R. The training and tested subsets of the data are set automatically within the package (43). We trained the random forest model with 1,000 trees and no specified maximum tree depth. The analysis presents the predictive accuracy of the model using the testing or ‘out-of-box’ data. Our response variable for the random forest analysis is the probability of CWD occupancy and thus the metric of evaluation used is the of out-of-box *R*^2^, higher values show better predictive ability of the model. Covariates with higher variable importance suggest they are more informative at explaining variability in occupancy rates. We used all habitat, soil, temporal, and spatial variables in the random forest analyses.

Preliminary analyses suggested that the temporal effect of year would dominate the variable importance followed by the latitude and longitude given the nature of the invasion. As we were particularly interested in soil or habitat features that were related with CWD occupancy, we fit separate random forest models for each year to determine if there were common factors that were important across years and how much variability in CWD occupancy was explained by these covariates across years.

## 3 Results

From 2005 to 2020, there were 18,421 samples collected for CWD surveillance in Kansas. There were 418 that were CWD positive, 17,861 that were CWD negative, and 142 that were indeterminant. There was a low of zero positive samples in 2006 to a high of 114 positive samples in 2019. Samples were from five species (Table 2). The majority (88%) of samples were from white-tailed deer (*Odocoileus virginianus*), 10% were from mule deer (*O. hemionus*), 1% from elk (*Cervus canadensis*), and cumulatively less than 1% of samples were from fallow deer (*Dama dama*), sika deer (*Cervus nippon*), or the species was unknown (Table 2).

**Table 2:** Number of CWD surveillance samples by species, shown with scientific names, number of positive CWD samples, indeterminant samples, and total number of samples.

Species	Scientific name	CWD positive	Indeterminant	Total
White-tailed deer	*Odocoileus virginianus*	319	129	16,247
Mule deer	*Odocoileus hemionus*	98	6	1,854
Elk	*Cervus canadensis*	0	0	236
Fallow deer	*Dama dama*	0	0	4
Sika	*Cervus nippon*	0	0	3
Species unknown		1	9	77
Total		418	142	18,421

### 3.1 Occupancy results

We limited the habitat, elevation, and soil covariate set to 15 by removing covariates that were highly correlated (*R* > 0.5). Using PCA we converted the habitat and soil covariates to fully orthogonal covariates and limited the principal components to ensure the cumulative proportion of variance explained was over 0.95, thus we ended up with 13 orthogonal covariates to explain habitat, elevation, and soil relationships. We compared models with different temporal patterns (Table 3). A linear trend across time and a model with splines with 5 degrees of freedom on year were similarly supported based on the WAIC values (Table 3). The linear model had a higher AUC (Table 3) and was considered the top model. The top model had an AUC of 0.89 suggesting very good model fit.

**Table 3:** Model comparison results from the dynamic occupancy model on CWD in Kansas from 2006–2020 to determine the best fitting temporal model.

Model	*k*	Delta WAIC	WAIC	AUC
Linear year trend	16	1.07	1562.43	0.891
Year spline df = 3	18	50.12	1611.48	0.887
Year spline df = 5	20	0.00	1561.37	0.876
Year spline df = 7	22	39.41	1600.77	0.878

All models included 13 principal components of the habitat and soil characteristics and a neighbor effect. The temporal models compare the degrees of freedom for the splines used on annual variability. The models are compared using WAIC where lower values are more parsimonious, the delta WAIC is shown to make comparisons easier. Additional model fit is shown as the occupancy adjusted AUC statistic, where higher AUC represents a better model fit. The number of parameters (k) are also proved.

**Figure 2:**
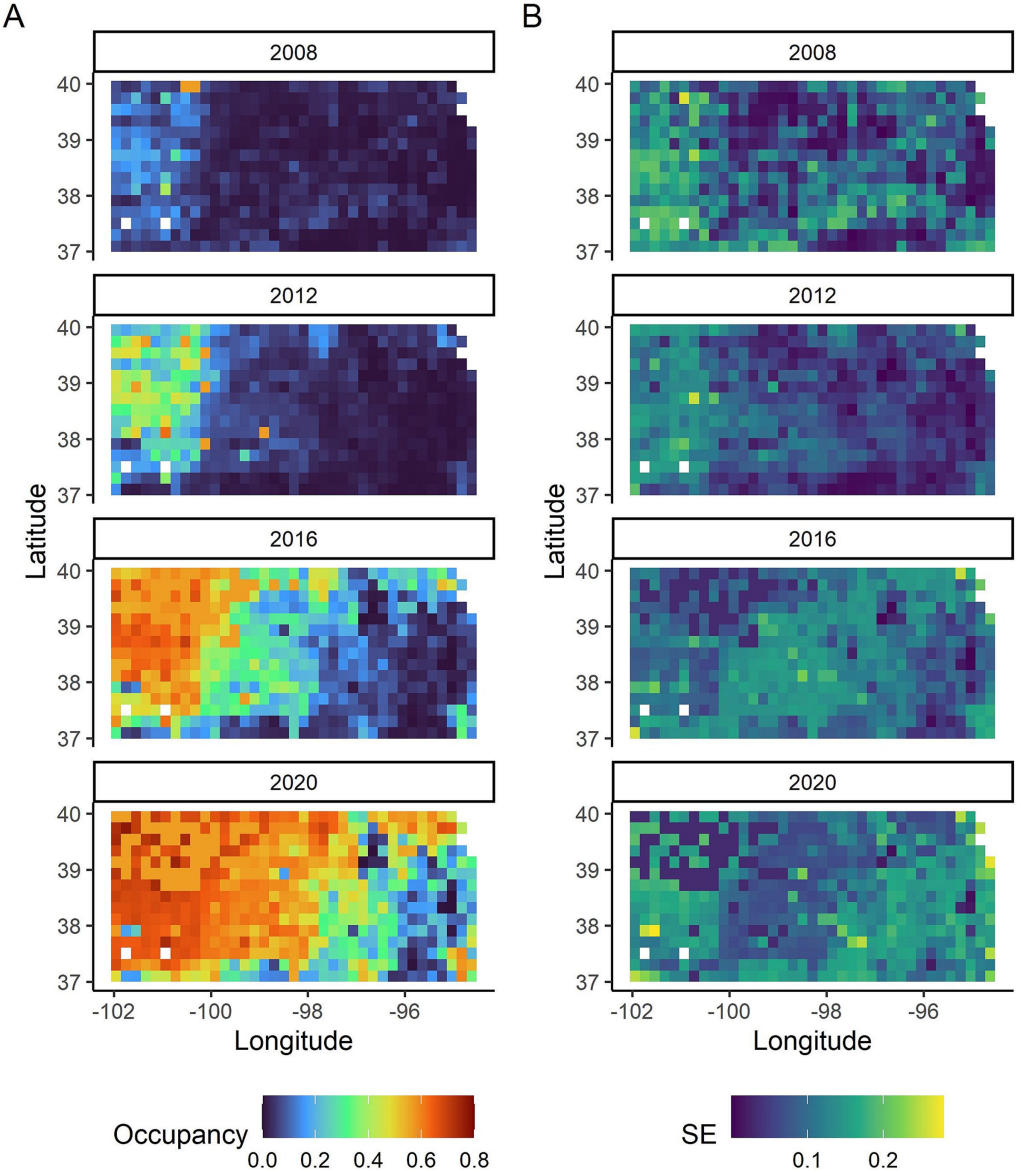
**(A)** CWD occupancy probabilities in Kansas shown every 4 years from 2008 to 2020. **(B)** The standard errors (SE) associated with the CWD occupancy probabilities in Kansas in the same years.

CWD occupancy varied considerably across space and time (Figure 2A). Generally, CWD occupancy was low early in the study with a low of 0.05 [95% credible intervals (CI): 0.001, 0.179] in 2008 to a high of 0.46 (95% CI: 0.04, 0.69) in 2020. The first few years had very few CWD positives and thus had greater uncertainty around the occupancy estimates than later years (Figure 2B). The probability of detecting CWD given it was present was 0.22 (95% CI: 0.19, 0.25).

### 3.2 Random forest results

Random forest models provide out of box *R*^2^ values describing the predictive accuracy of the model. Although the *R*^2^ values for individual years varied, ranging from 0.4 when there were very few cases of CWD in the initial years to considerably better (0.86–0.93) in the latter years, the random forest out of box *R*^2^ value for all of Kansas and all years 2006–2020 was 0.96, suggesting the covariates considered were able to explain a large majority of the variation in CWD occupancy and have very good predictive accuracy. The annual variability (i.e., year effect) had, by far, the greatest variable importance (0.11). The next highest variable importance was less than 1/14th of the importance of the year effect (variable importance of elevation = 0.008), suggesting that annual variability explained most of the CWD occupancy in Kansas.

**Figure 3:**
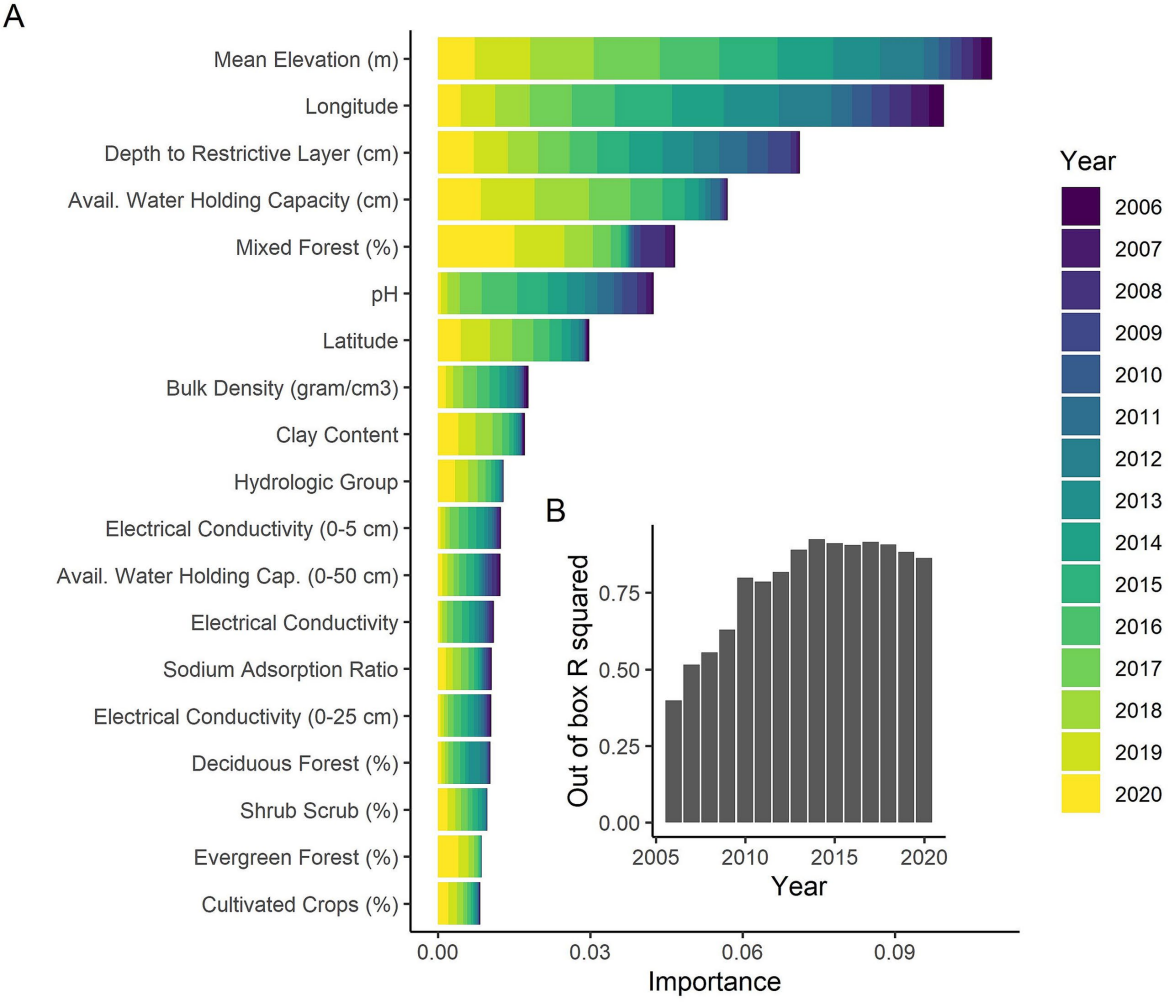
**(A)** The variable importance values from the random forest analyses separately conducted on each year of data in Kansas from 2006 to 2020. Larger variable importance values suggest the covariate explain more variability in the response variable, in this case CWD occupancy. The covariates are shown in decreasing order of cumulative variable importance across all years. The top 20 covariates across all years are shown. The years are represented by colors. **(B)** The out of box R-squared values from the random forest analyses by year. Higher R-squared values suggest that the model is better explaining the variability in the system.

**Figure 4:**
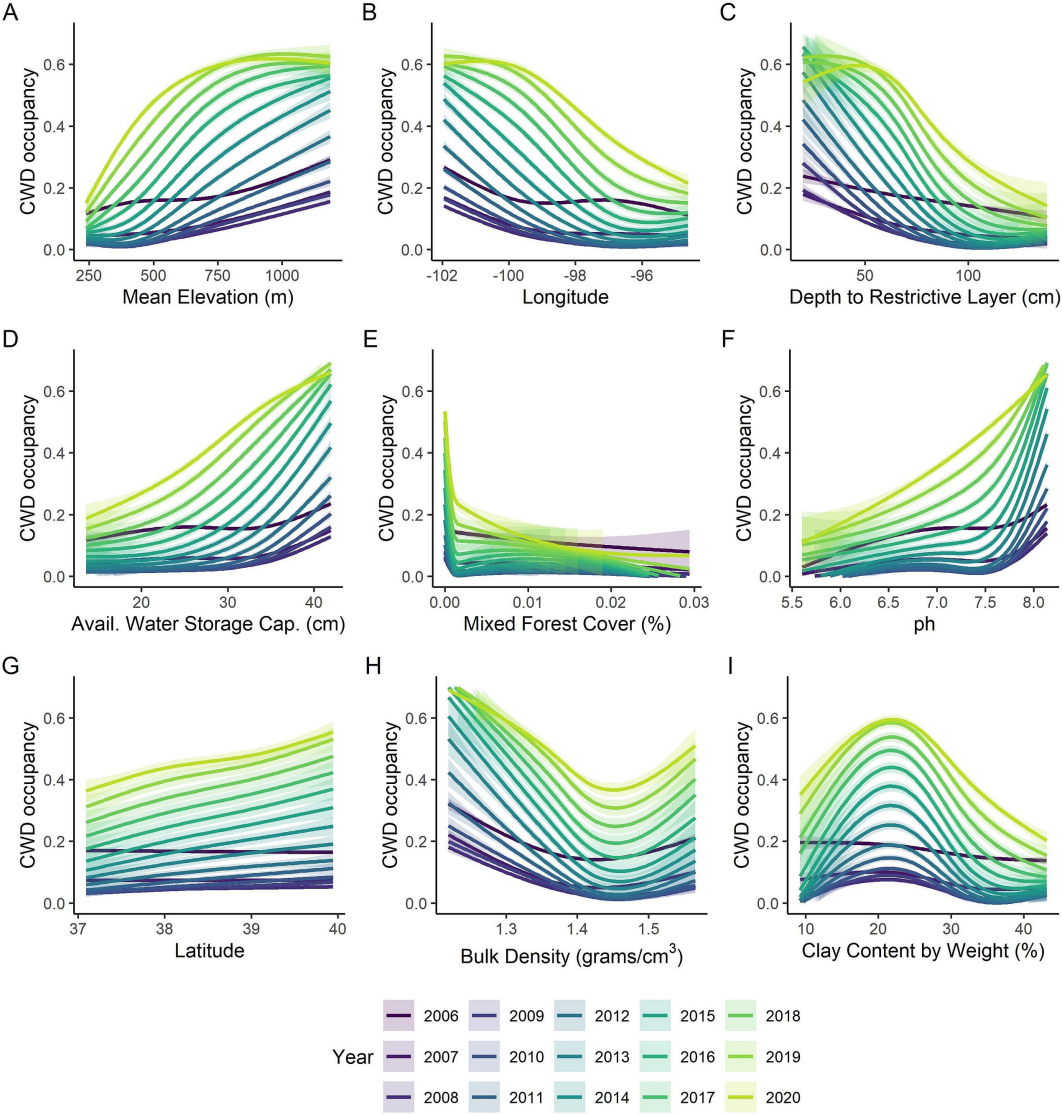
Marginal plots of relationships between **(A)** elevation, **(B)** longitude, **(C)** depth to restrictive layer (cm), **(D)** available water storage capacity (cm), **(E)** percent cover of mixed forest, **(F)** pH, **(G)** latitude, **(H)** Bulk density (grams/cm^3^) and **(I)** percent clay by weight and the probability of CWD occupancy in Kansas shown across years 2006–2020 (shown by color). The lines represent the smoothed marginal relationship from the yearly random forest analyses, the shaded regions represent the 95% confidence intervals.

We conducted random forest analyses separately by year to look more closely at impacts of habitat and soil covariates as well as to examine how well the models fit by year. Mean elevation was the most important variable across all years (Figure 3A) with higher occupancy probabilities being associated with higher elevations (Figure 4A). Longitude and latitude were also important explanatory variables for CWD occupancy (Figure 3A) as they help describe the spatial spread over time (Figures 2, 4B, H). Shallower depths to restrictive soil layers were associated with higher CWD occupancy (Figure 4C). Higher available water storage capacity related to higher CWD occupancy (Figure 4D). When there was no (0%) mixed forest coverage that tended to relate to higher CWD occupancy (Figure 4E) and which was a more important factor in early and late years (Figure 3A). Lower soil temperature regimes tended to correlate with higher CWD occupancy (Figure 4F) and this was a more important factor in later years of the study (Figure 3A). Higher pH levels also tended to correlate with higher CWD occupancy (Figure 4G), which was more important earlier in the study (Figure 3A). Higher CWD occupancy was also associated with 15–25% of clay composition in the soil (Figure 4I) although this relationship was less important than other covariates already described (Figure 3A). Of the 77 soil, habitat, and elevation covariates that we examined, the nine described here explained the most variability (Figure 3A). The out of box R-squared values were higher in later years than earlier years (Figure 3B). Earlier years had considerably fewer CWD positive cases and thus most sites were likely free of CWD early in the study (Figure 2). Spatial pattern of different covariates evaluated in the study is present in Supplementary Files 1, 2.

## 4 Discussion

The spatiotemporal occupancy patterns of CWD-infected deer in Kansas (Figure 2) illustrate the geographic expansion of CWD in the state, progressing from the northwest corner toward the central and eastern areas over the years. This pattern largely reflects a natural spatial progression of CWD in the state due to deer movement, rather than a pattern of accidental introductions over time into new areas aided by hunters or other agents. If the latter were the case, it would have likely resulted in a non-uniform spatial pattern with prominent, heterogeneous spatial foci of higher occupancies. The initial infections in Kansas likely originated from deer that migrated from the western states of Colorado and Wyoming, and Nebraska to the north, where the disease was established a number of years prior (44, 45). The number of positive CWD cases in the central and eastern side of the state remains low at the present time, despite higher prevalence of CWD infected deer in Missouri to the east [e.g., (46)]. Prevention and/or management strategies such as targeted culling when new cases occur in the central and eastern region Kansas may prevent the further spread of CWD in the state.

Areas in the state with higher soil pH values (alkaline) are associated with higher CWD occupancy. Additionally, CWD occupancy changed non-linearly with increasing clay content in the soil, peaking slightly above 20% and gradually declining beyond that level, with an optimal range between 18 and 25% by weight. These observations are similar to those made in previous studies [e.g., (28, 29, 47)]. Dorak et al. (28) found that the potential exposure to prions in Illinois soils increases when the soil pH is above 6.6 and the clay content is below 18%, which aligns with our current findings. The similarity is remarkable, given the differences between the two studies in terms of modeling approaches and soil environmental datasets. We found lower depths to restrictive layer in the soil were associated with higher CWD occupancy, suggesting that the areas with soils that have shallower distance to claypan, or bedrock are potentially more suitable for prion persistence and subsequent transmission to deer. This aligns with our observation that regions with soils possessing higher available water storage (AWS) capacity also exhibit higher CWD occupancy. Soils with shallow restrictive layers generally have lower AWS capacity. Higher bulk density indicates more compacted soil with less pore space, which can affect prion persistence, bioavailability, and movement assisted by wind and water. We observed that CWD occupancy is non-linearly associated with bulk density over a narrow range of values between 1.2 and 1.6 g/cm^3^. Areas with lower bulk density values favor higher CWD occupancy, but this occupancy quickly declines over a short range before increasing again.

The heterogeneities in soil physiochemical properties that affect CWD occupancy are an indication of landscape-level factors that can be targeted for management. Understandably, it is impracticable nor advised to amend soil properties on such large scales to prevent CWD transmission. However, this understanding allows us to focus management strategies on 20 km^2^ areas, or contiguous groups of 20 km^2^ areas, which are likely to have higher CWD occupancy and also possess high-risk soil properties. One previously proposed approach is targeted culling in areas with higher deer density and riskier soil physiochemical properties (28, 29, 48). However, the practice of targeted or local culling must be continuously evaluated for its effectiveness in reducing CWD infection among cervids (49, 50). Carcass removal from the landscape in areas where CWD is present and soil properties are high risk, and restricting feeding or baiting in these areas to minimize deer congregation and thereby concentrated prion environmental deposition have also been proposed as management actions (cite). In naïve, uninvaded high-risk areas, wild and captive cervid movement could be restricted along with restrictions placed on hunting activity and carcass disposal, among others, which may prevent the dispersal and invasion of CWD prions. The diagnostic detection of CWD prions in environmental samples has become more accessible and standardized with the recent adaptation of the RT-QuIC assay. This advancement enables the establishment of monitoring programs to track CWD prion levels across different management zones and monitor prion movement, for instance through watersheds or other catchment areas.

The environmental correlations observed in this study may represent important relationships with CWD occupancy in the broader region; however, it should be noted that some of these associations might be spurious and/or related with the happenstance of the invasion. For instance, we observed that higher elevations were related to higher CWD occupancy, but this is likely due in large part to the fact that there is a higher concentration of cases in the relatively high-altitude western portion of Kansas where initial invasion likely came from Colorado and Wyoming. Infected cervids are known to be present in lower elevation area as in the case of their wider presence in Illinois and Missouri, in relatively flat terrains. Therefore, elevation at the scale of our analysis may not be a good predictor of future CWD occurrence within Kansas, but more of an explanation of where it came from. Similarly, mixed forest cover was the only habitat-specific covariate that was suggested to be important for CWD occupancy in Kansas. More mixed forest cover was less associated with CWD occupancy. However, the percent of mixed forest cover only ranged from 0 to 3% cover within the 20 km^2^ grids we considered across the state. This may be another example of instances where the happenstance of the invasion is suggesting a relationship that is not necessarily a driver of CWD occurrence in Kansas. Future studies evaluating the association of environmental covariates with CWD, and other wildlife diseases may benefit from using derived remote sensing variables of factors that affect land cover land use change [e.g., (51–53)] in addition to the crude estimates as used in the present study.

The 20 km^2^ spatial units for analysis in this study is somewhat artificial and it is not the scale in which deer are managed in the state of Kansas—which are several kilometers wide/deep, and they are based on road networks for borders (54). Although strictly not reflective of the home ranges of white-tailed-and mule deer in the region, which varies by season, region, and sex, and can range from 1 to 5 km^2^ in Texas (55–57) the spatial unit we used for modeling the occupancy status allowed us to potentially account for uncertainties associated with harvest locations reported by sample submitters, as well as deer movement during the hunting season. Compared to the current deer management units, the finer, more uniformly sized 20 km^2^ spatial units we used in the present study are potentially more suitable when it comes to deciding, implementing CWD management strategies, such as localized and targeted culling. This is particularly valuable in the newly invaded areas in the central and eastern sides of the state, which may not be predictable or visible at coarser scales of spatial analysis. Also, in the present work, we did not allow detection probability to vary, however, it would be possible with a modification to our current approach to estimate detection probabilities separately, for instance if differences are expected for different sources of surveillance or diagnostic methods, which may help inform management and/or surveillance decisions [e.g., (58)].

The probability of detecting CWD in our study exceeded our expectations (average detection probability given CWD was present was 0.22), given the inherent challenges of modeling spatiotemporal patterns for wildlife diseases (58, 59), and it was also higher than that reported in similar studies. For instance, our own studies assessing wildlife rabies in North America, which incorporated various landscape-level covariates similar to the present study, typically reported detection rates ranging from 0.02 to 0.15 at the higher end (58–60), compared to up to 0.83 in the present study. The standard errors noted for occupancies in the present study are low and did not raise concerns. Further, the AUC values for all the models considered in the study were within 15% of each other and close to 0.9, suggesting an excellent model fit (Table 3). This indicates that the random effect terms and the inclusion of fixed environmental covariates in the study adequately captured the variability in CWD data.

One of the epizootiological aspects of cervid CWD that is not yet fully understood is the potential prevalence of CWD prions in various scavenger and predator species, along with their exact role in the disease’s spread (61, 62). The soil properties observed in this study that influence CWD occupancy also indirectly affect various ecological parameters, including nutrient availability, plant growth rate, and vegetation density. These factors, in turn, influence the habitat quality and suitability for different scavenger species, affecting their composition, diversity, density, and movement patterns across the landscape. Together, these factors potentially impact CWD dispersal in the environment. Future studies that evaluate the role of scavengers in CWD epizootiology will benefit by considering the underlying landscape-level soil characteristics. For instance, spatially informed sampling of scavenger species may be conducted in areas where certain soil conditions indicate potential for higher CWD prion levels. In conclusion, this study further shows that occupancy class models are a valuable tool in wildlife disease spatiotemporal modeling and for evaluating environmental drivers of such diseases. The spatiotemporal patterns and environmental factors identified in the present study will be useful in managing this disease in Kansas and potentially other geographic regions.

Some potential defects in our models and the resulting occupancy predictions must be mentioned. The choice of spatial extent (20 km^2^ grid) for analysis could impact our predictions; too coarse of a scale would result in overestimation of occupancy, whereas too fine a resolution could have the opposite effect. We also assumed that a site’s occupancy status remains constant over the annual time step, which may be unrealistic, particularly in areas with fewer CWD-positive deer harvested, especially since surveillance was limited to the deer hunting season, lasting approximately 7–8 weeks each year. We also assume that our surveillance samples all have equal probability of detection, which may or may not be the case and needs a second assessment with different model specifications.

## Data Availability

The original contributions presented in the study are included in the article/Supplementary material, further inquiries can be directed to the corresponding author.

## Supplementary material

SUPPLEMENTARY FILE 1 Spatial pattern of different covariates evaluated in the study.

SUPPLEMENTARY FILE 2 Spatial pattern of different covariates evaluated in the study.
