# Supplementary figures and images for: Spatiotemporal occupancy patterns of chronic wasting disease
Source: Front Vet Sci. 2024 Nov 20;11:1492743. doi: 10.3389/fvets.2024.1492743 (PMC11615082; doi:10.3389/fvets.2024.1492743)

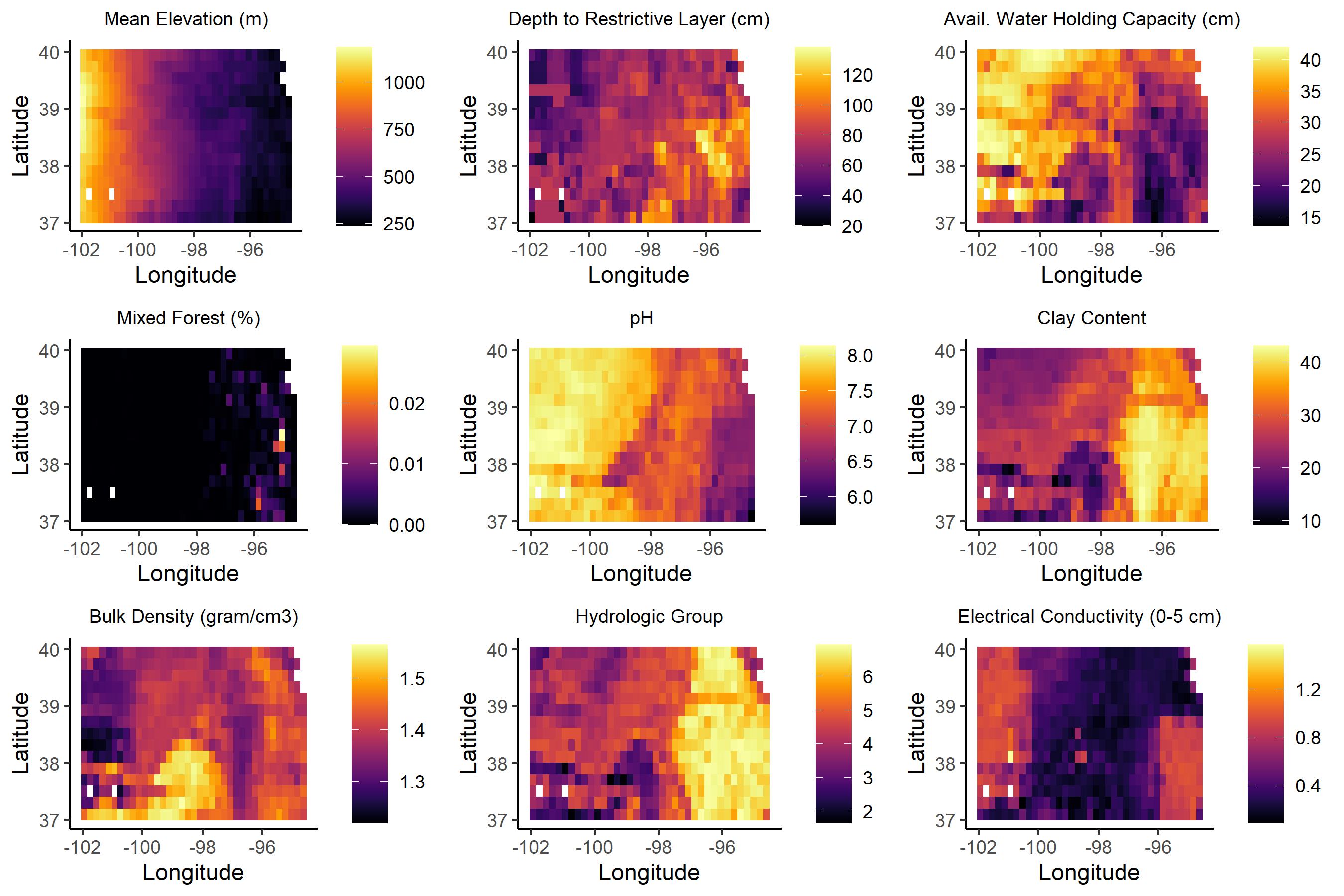

Supplement: SUPPLEMENTARY FILE 1 — Spatial pattern of different covariates evaluated in the study. [file Image_1.JPEG]

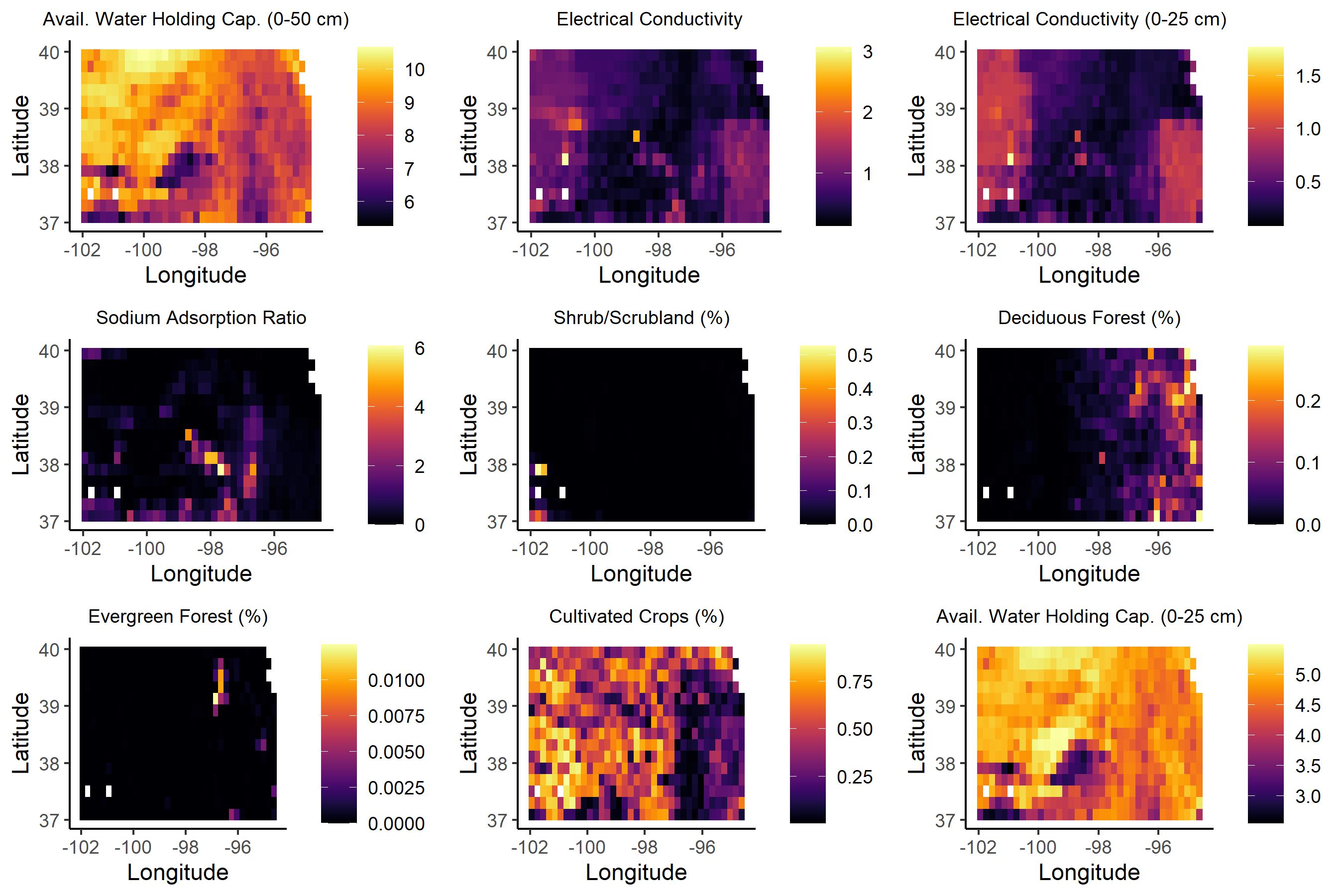

Supplement: SUPPLEMENTARY FILE 2 — Spatial pattern of different covariates evaluated in the study. [file Image_2.JPEG]
